# Syntaxin 1: A Novel Robust Immunophenotypic Marker of Neuroendocrine Tumors

**DOI:** 10.3390/ijms21041213

**Published:** 2020-02-12

**Authors:** Bence Kővári, Sándor Turkevi-Nagy, Ágnes Báthori, Zoltán Fekete, László Krenács

**Affiliations:** 1 Department of Pathology, University of Szeged, Szeged 6725, Hungary; turkevi-nagy.sandor@med.u-szeged.hu (S.T.-N.); bathori.agnes87@gmail.com (Á.B.); z0li950912@gmail.com (Z.F.); 2 Laboratory of Tumor Pathology and Molecular Diagnostics, Szeged 6726, Hungary; krenacsl@vipmail.hu

**Keywords:** neuroendocrine neoplasia, neuroendocrine tumor, neuroendocrine carcinoma, immunohistochemistry, syntaxin 1

## Abstract

Considering the specific clinical management of neuroendocrine (NE) neoplasms (NENs), immunohistochemistry (IHC) is required to confirm their diagnosis. Nowadays, synaptophysin (SYP), chromogranin A (CHGA), and CD56 are the most frequently used NE immunohistochemical markers; however, their sensitivity and specificity are less than optimal. Syntaxin 1 (STX1) is a member of a membrane-integrated protein family involved in neuromediator release, and its expression has been reported to be restricted to neuronal and NE tissues. In this study, we evaluated STX1 as an immunohistochemical marker of NE differentiation. STX1, SYP, CHGA, and CD56 expression was analyzed in a diverse series of NE tumors (NETs), NE carcinomas (NECs), and non-NE tumors. All but one (64/65; 98%) NETs and all (54/54; 100%) NECs revealed STX1 positivity in at least 50% of the tumor cells. STX1 showed the highest sensitivity both in NETs (99%) and NECs (100%) compared to CHGA (98% and 91%), SYP (96% and 89%), and CD56 (70% and 93%), respectively. A wide variety of non-NE tumors were tested and found to be uniformly negative, yielding a perfect specificity. We established that STX1 is a robust NE marker with an outstanding sensitivity and specificity. Its expression is independent of the site and grade of the NENs.

## 1. Introduction

Neuroendocrine (NE) cells comprise a cellular network integrating the nervous and endocrine systems. They are found in virtually all organs, and the most common are in the gastrointestinal and lower respiratory tracts. The NE cells secrete biogenic amines and peptide hormones regulating a wide variety of functions into the bloodstream. Tumorous growths of NE cells are collectively referred to as NE neoplasms (NENs). The bioactive substances secreted by neoplastic NE cells can lead to distinct clinical syndromes. Although the NENs may follow an indolent clinical course, a significant number of patients are diagnosed with advanced disease. Although there are many organ-specific differences in tumor biology and prognostic factors among NENs of different localizations, according to the recommendations of the International Agency for Research on Cancer (IARC) and World Health Organization (WHO) expert consensus proposal, the terminology of NENs should be uniformized in the future. Therefore, the currently substantially differing organ-specific classification schemes of NENs will be potentially revised and harmonized within the next edition of each WHO Blue Book [1,2]. This proposal separates well-differentiated and poorly differentiated NENs. The well-differentiated NENs are also referred to as NE tumors (NETs), while poorly differentiated highly malignant NENs are also designated as NE carcinomas (NECs) ([2,3] (pp. 210–214), [4] (pp. 18–19)). The histopathologic diagnosis of NENs is based on the typical cytomorphological and architectural features of these tumors; nevertheless, in atypical and especially poorly differentiated cases, immunophenotyping is inevitable [5,6,7]. Rare tumors with two distinct NE and non-NE cell populations, in which either component represents at least 30%, are defined as a mixed neuroendocrine-non-neuroendocrine neoplasm (MiNEN) [4] (pp. 16–20). These cases may be difficult to recognize, particularly when they are poorly differentiated, and their accurate classification also requires immunohistochemistry (IHC) evaluation.

NE cells share common antigens characteristic of NE differentiation, which can be utilized as immunophenotypic markers. Some of these are rather obsolete, such as the neuron-specific enolase (NSE) and protein gene product 9.5 (PGP9.5), which are very sensitive, but seriously lack specificity [6,8]; others, such as synaptophysin (SYP), chromogranin-A (CHGA), CD56, and insulinoma-associated protein 1 (INSM1), are more reliable, but still show disadvantages when used as individual markers [8,9,10,11,12,13,14,15].

Syntaxin 1 (STX1) is a member of a membrane-integrated nervous system-specific protein superfamily involved in the neuromediator release from synaptic vesicles [16,17,18]. STX1 plays a crucial role in ion channel regulation and synaptic exocytosis [16,17,18]. Two STX1 isoforms, HPC-1/syntaxin 1A and syntaxin 1B, are thought to have similar functions in the exocytosis of synaptic vesicles and show a very high homology [19,20]. Additionally, it has been reported that STX1 is associated with chromaffin granules in the adrenal medulla [21] and expressed in alpha, beta, and delta cells of pancreatic islets [22,23]. Using a Web-based in silico biomarker analysis, syntaxin 1A protein expression has been found to be restricted to NE cells [24]; however, this finding was not further evaluated.

Since STX1 represents a promising NE marker and has not yet been comprehensively tested in diagnostic pathology, we performed a retro- and prospective IHC study on a diverse series of benign and malignant tumors, in order to establish its utility in the immunophenotyping of NENs.

## 2. Results

**Figure 1:**
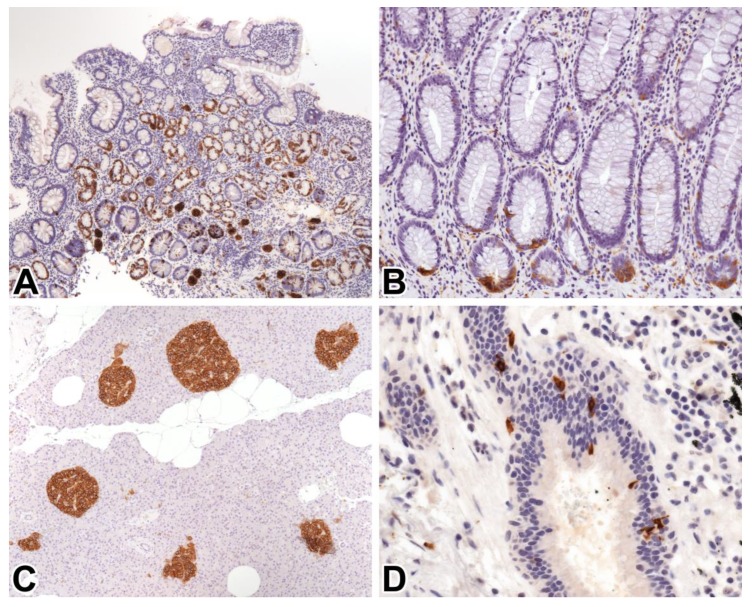
Syntaxin 1 (STX1) immunoreactivity in non-neoplastic tissues. (**A**) Nodular neuroendocrine (NE) cell hyperplasia in autoimmune metaplastic atrophic gastritis, 10x; (**B**) NE cells in normal colonic mucosa, 20x; (**C**) Langerhans islets in normal pancreatic tissue, 10x; (**D**) NE cells in normal bronchial mucosa, 40x.

Virtually all normal NE cells and hyperplastic NE lesions showed strong membranous and weak to moderate cytoplasmic STX1 staining (Figure 1). Thyroid follicular cells, adrenocortical tissue, and non-NE cells, including gastrointestinal mucosa, exocrine pancreas, liver, kidney, lymphoid tissue, and skin, showed no STX1 positivity. Neuronal tissue in the brain and peripheral nerves, including hypertrophic myenteric plexus in appendicitis specimens, revealed consistent STX1 staining. The results obtained with STX1 in NE and neuroepithelial neoplastic samples are summarized in Table 1. 

**Figure 2:**
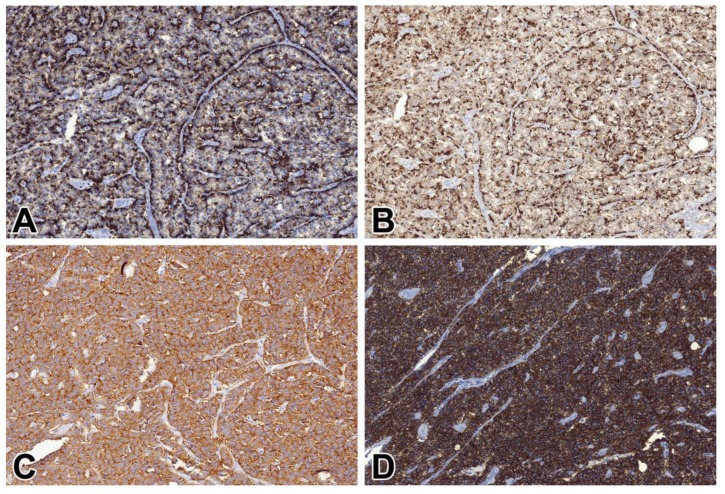
CD56, CHGA, SYP, and STX1 immunoreactivity in a pancreatic non-functioning NE tumor (NET) grade 2. (**A**) CD56, 20x; (**B**) CHGA, 20x; (**C**) SYP, 20x; (**D**) STX1, 20x.

**Figure 3:**
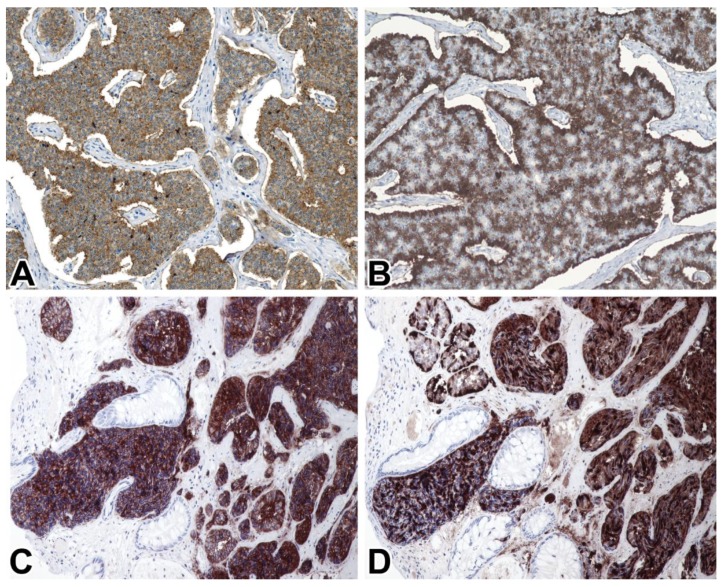
STX1 (**A**,**C**) vesicular monoamine transporter 1 (VMAT1), (**B**) and glucagon-like peptide 1 (GLP1) (**D**) immunoreactivity in a small intestinal enterochromaffin (EC) cell NET grade 2 (**A**,**B**) and a rectal L cell NET grade 1 (**C**,**D**) 20x.

**Figure 4:**
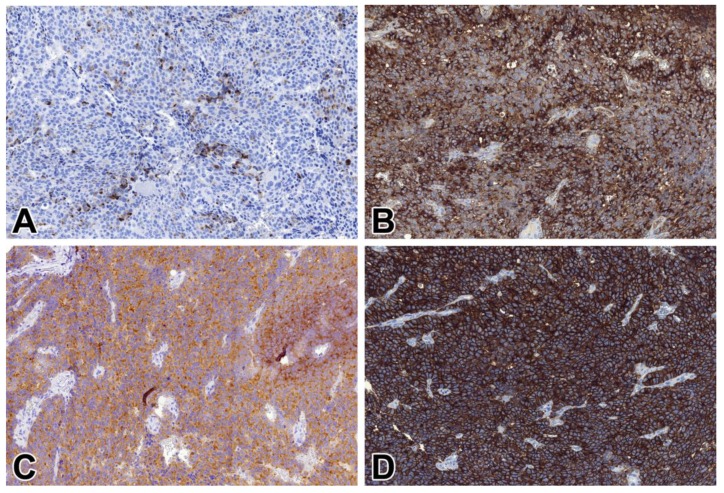
CD56, CHGA, SYP, and STX1 immunoreactivity in a pancreatic large cell NE carcinoma (NEC). (**A**) CD56, 20x; (**B**) CHGA, 20x; (**C**) SYP, 20x; (**D**) STX1, 20x.

**Figure 5:**
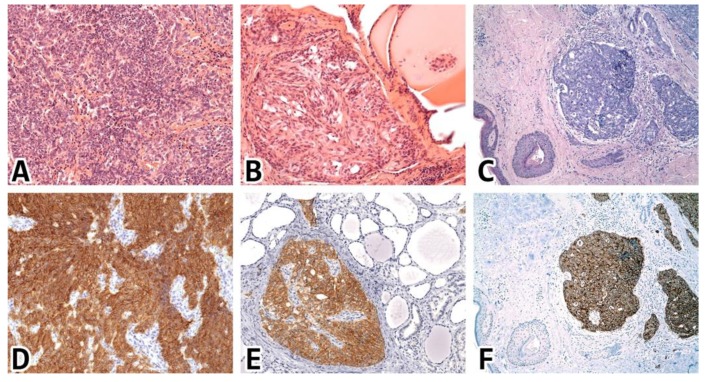
STX1 immunoreactivity in various types of NECs. (**A**–**C**) Hematoxylin-eosin stainings; (**D**–**F**) STX1 immunohistochemistry (IHC) reactions; (**A**,**D**) small cell NEC of the lung, 20x; (**B**,**E**) medullary thyroid carcinoma, 20x; (**C**,**F**) Merkel cell carcinoma of the skin, 10x.

All but one (99/100, 99%) cases of NENs, including metastatic NETs, proved to be STX1-positive (Figure 2). Regarding specific subsets of gastrointestinal NETs, vesicular monoamine transporter 1 (VMAT1)-positive EC cell NETs; VMAT1-negative, autoimmune gastritis-associated NETs consistent with ECL cell NETs; and even rectal glucagon-like peptide 1 (GLP1)-positive L cell NETs, were consistently positive (Figure 3). At least 50% of the tumor cells were labeled with a moderate to strong intensity, but in 92% of cases, the positivity rate was more than 85%. The STX1 staining intensity showed no correlation with the mitotic activity, Ki-67 labeling index, or tumor grade. The single STX1-negative case represented a grade 2 CHGA-positive NET of the major duodenal papilla. All small and large cell NECs were consistently STX1-positive, mostly with intense diffuse staining, regardless of the anatomical site (Figure 4). Furthermore, special types of NECs, such as Merkel cell and medullary thyroid carcinomas, similarly showed uniform strong diffuse immunoreactivity for STX1 (Figure 5).

The staining pattern was usually both membranous and cytoplasmic. The ratio of these two patterns varied considerably, from predominantly cytoplasmic to complete diffuse membranous. Membrane staining was typically complete in NETs, although in some cases with an acinar architecture, a basolateral membranous staining pattern was revealed. In some NECs, predominantly aberrant incomplete membrane staining was noticed. In comparison to common NE IHC markers, STX1 showed the highest sensitivity both in NETs (99%) and NECs (100%), which was followed by CHGA (98% and 91%), SYP (96% and 89%), and CD56 (70% and 93%), respectively (Table 2). The four applied markers detected a significantly different proportion of positive cases regarding gastrointestinal NETs (*p* < 0.000001), gastrointestinal NECs (*p* = 0.01), and NECs in general (*p* = 0.007). In terms of pancreatic NETs, no statistically significant level of such an association was seen.

In six tumors with mixed NE and non-NE components, including those with only minor NE components and those qualifying as MiNENs, the STX1 expression topographically always colocalized with the morphological features of NE differentiation (Table 1), while the exocrine component showed no STX1 positivity.

STX1 expression was generally absent in conventional carcinomas. In a subset (16/215, 7%) of conventional (non-NE) neoplasia, a discrete (<10%) and scattered intratumoral STX1-positive cell population was noticeable without a definite NE morphology. The expression of at least one further (CD56, SYP, or CHGA) NE marker was also detected in all these cases. The majority (10/13, 77%) of the hypercellular variant (type B) of mucinous breast carcinoma cases and one case of ductal carcinoma demonstrated STX1 positivity, usually in more than 60% of the tumor cells (Table 3).

**Figure 6:**
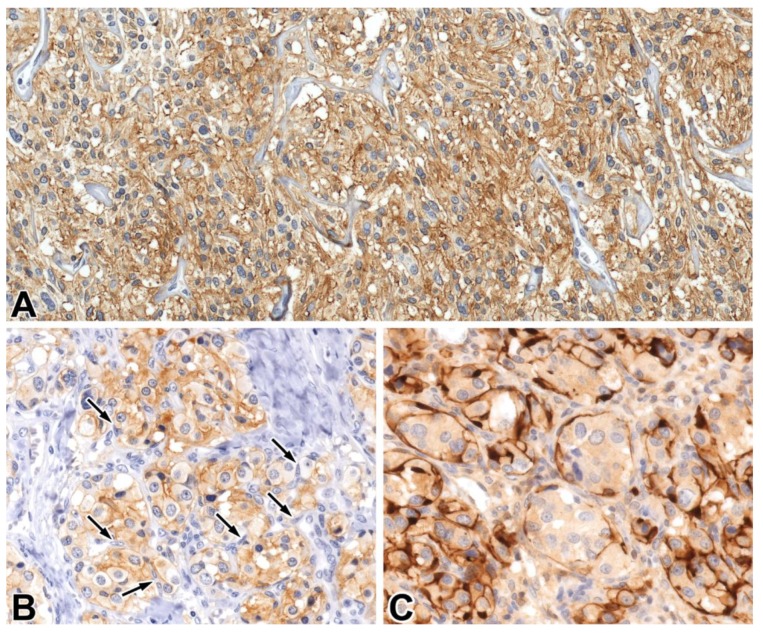
STX1 (**A**,**B**) and S100 (**C**) immunoreactivity in pheochromocytomas, for which the arrows indicate sustentacular cells (**B**) The latter cells were visualized with an S100 IHC reaction. (**A**): 20x, (**B**,**C**): 40x.

All 20 (100%) pituitary adenoma cases and 14/16 (88%) of pheochromocytoma cases showed STX1 positivity (Table 1, Figure 6). The latter tumor group included 12 cases with diffuse staining and two cases with 30% tumor cell positivity. The two STX1-negative medullary chromaffin cell-derived tumors represented one benign and one malignant pheochromocytoma case. In three pheochromocytomas, faint and focal STX1 positivity was also exhibited by the sustentacular cells. Endocrine neoplasias, including adrenocortical adenoma and adrenocortical carcinoma, parathyroid adenoma, papillary thyroid carcinoma, and follicular thyroid adenoma cases, were consistently STX1-negative (Table 3).

All but one neuroectodermal/neuroepithelial tumor samples revealed strong diffuse STX1 positivity in neuroblasts and ganglion cells (Table 1), whereas less than 10% of tumor cells were positive in one medulloblastoma case. Where it was present, the neuropil component also showed a moderate expression. The Schwann cell component of the ganglioneuromas, and all peripheral nerve sheet tumor cases, proved to be STX1-negative (Table 3). Scattered STX1-positive tumor cells were found in 2/2 Ewing family tumor cases studied.

## 3. Discussion

There are several IHC NE markers used in diagnostic pathology. To use these markers responsibly, one should be aware of their advantages and limitations [12,13,14,15]. Currently, CD56, CHGA, and SYP represent the most widely used NE markers. CD56 is highly sensitive to NE cells [4]; however, it is also detectable in a heterogeneous group of non-NE neoplasms, including nephroblastoma, neuroblastoma, myeloid, lymphoid, and plasma cell tumors, as well as in hepatocellular, renal cell, ovarian, endometrial, and thyroid carcinomas [25,26]. Therefore, it is unreliable if used as a single marker. CHGA constitutes a secretory granule protein of the NE, adenohypophyseal, and parathyroid cells [27,28]. As an IHC NE marker, CHGA shows a good sensitivity; nevertheless, some NE cells, such as Merkel cells and hindgut-derived NE cells [29], may demonstrate significantly weaker staining or can lack CHGA immunoreactivity. Furthermore, the strong cytoplasmic expression seen in most NE cells may be weak or absent in NECs, due to the low density of mature secretory granules [30,31]. SYP is an integral membrane calcium-binding glycoprotein of synaptic vesicles. It is a sensitive marker usually expressed in the cytoplasmic microvesicles of neuronal tumors, NENs, and neuroectoderm-derived small blue cell tumors, as well as in many endocrine tissues, such as normal and neoplastic pituitary and parathyroid glands and adrenocortical tissue [32,33,34].

In this study, we performed a comprehensive IHC analysis in an extensive series of benign and malignant tumors to evaluate STX1 as an IHC NE marker. Using a well-characterized monoclonal mouse antibody, we found that STX1 represents an impressive robust NE marker, with a sensitivity of 99% in NETs and 100% in NECs, outperforming other common NE markers, such as SYP (96% and 89%), CHGA (93% and 91%), and CD56 (70% and 93%), which was proven to be statistically significant regarding gastrointestinal NETs and NECs. Normal NE cells in different organs, as well as pulmonary, gastrointestinal, and pancreatic NETs and NECs, were likewise positive. Therefore, the STX1 expression of NENs seems to be unrelated to the anatomical site. In contrast to the frequently negative CHGA staining in rectal and appendiceal L-cell NETs [9,10,11], STX1 was consistently positive in all such cases studied. Pheochromocytomas and paragangliomas were also almost consistently positive. STX1 was uniformly expressed in all NECs, regardless of the morphological or clinicopathological subtype, including small and large cell NECs, as well as in Merkel cell carcinomas and medullary thyroid carcinomas. In contrast to the sometimes faint or dot-like cytoplasmic expression of SYP and CHGA, the STX1 revealed crisp membranous and strong cytoplasmic staining in the majority of cases studied, which makes the evaluation straightforward. Concerning the specificity of STX1, many endocrine tumors known to express either CHGA or SYP, such as parathyroid and adrenocortical neoplasms [35], were consistently negative for STX1. As further evidence of the excellent specificity of STX1, a broad spectrum of non-NE tumors, including various types of carcinomas, were consistently negative. In MiNENs and carcinomas with focal NE components, the STX1 immunostaining was restricted to the NE areas, as was confirmed by other NE markers. A caveat is that in 7% of conventional (non-NE) neoplasia, a discrete (<10%) and scarcely scattered STX1-positive cell population was present without morphological features of NE differentiation. In our opinion, by correlating the IHC stains to the histomorphology, these infrequent positive cells should not cause any diagnostic problem, and such reactions should be interpreted as negative. Although STX1 was also expressed in neural tumors, considering the rather distinct presentation of these neoplasms, the differentiation appears to be straightforward.

INSM1 is a recently described IHC NE marker [36], demonstrating nuclear localization. It has been studied in the NENs of many anatomical regions, including the lung, head and neck, central nervous system, prostate, skin, and pancreas, but, according to the reported series, the INSM1 reveals a lower sensitivity [13,36,37,38,39,40,41] than STX1 in our series. Although a direct comparison of the two markers is warranted in future studies, based on the literature data and results of the present study, STX1 outperforms INSM1 in terms of its specificity and sensitivity.

In keeping with the frequent NE differentiation of hypercellular (type B) mucinous breast carcinomas, a small cohort of these tumors were also stained positive for STX1, while breast cancer types infrequently expressing NE markers, such as hypocellular (type A) mucinous, conventional invasive ductal, and lobular carcinomas, were negative. Due to the limited number of breast tumors included in this study, additional investigations of breast cancer are warranted.

## 4. Materials and Methods 

### 4.1. Samples Studied

All samples were assigned by the Department of Pathology, University of Szeged, and the Laboratory of Tumor Pathology and Molecular Diagnostics, Szeged, Hungary, and represented formalin-fixed paraffin-embedded (FFPE) tissues.

To study STX1 in non-neoplastic tissues, normal thyroid (8 samples), parathyroid (11 samples), skin (2 samples), pancreas (5 samples), adrenal gland (9 samples), and brain tissue (5 samples), as well as appendectomy specimens from acute appendicitis (5 cases), were evaluated. Hyperplastic NE lesions, such as linear and nodular NE cell hyperplasia in autoimmune metaplastic atrophic gastritis (10 cases) morphologically consistent with ECL cell hyperplasia [42] and one case of pancreatic nesidioblastosis, were also included. In tumor samples, peritumoral non-neoplastic NE cells, including gastric antral and oxyntic mucosal, as well as intestinal NE cells, pancreatic Langerhans islets, bronchial NE cells, and cutaneous Merkel cells, were also assessed for STX1 expression, where they were present. 

To evaluate the specificity and sensitivity of STX1 in neoplastic conditions, altogether, 398 cases of various non-NE and NE neoplasms were studied (Table 1) in either whole tissue sections or tissue microarrays (TMAs). Cases with potential diagnostic pitfalls, for example, frequently CHGA-negative L cell NETs (three rectal and one appendiceal), were also included. In 6/215 cases of predominantly non-neuroendocrine carcinomas, a component showing morphological features of neuroendocrine differentiation was also present. In four (one colonic and one diffuse gastric adenocarcinoma, one poorly differentiated squamous cell, and one adenocarcinoma of the lung) cases, the neuroendocrine component represented 10%–30% of the tumor, while in two (one non-mucinous breast and one poorly differentiated gastric adenocarcinoma) cases, the NE component exceeded the MiNEN defining 30% [5] (pp. 18–19). TMA blocks were constructed with the manual TMA builder instrument (Histopathology Ltd., Pécs, Hungary), as previously published [43]. Each tumor case in TMA blocks was represented with at least two cores (central and peripheral regions) of 2 mm. To compare the proportion of positive cases by the applied markers, Fisher’s exact test was performed.

All NENs were diagnosed and graded according to the WHO Blue Book [1], corresponding to their anatomical location.

The study was performed in agreement with the guidelines of the Declaration of Helsinki for human medical research and was ethically approved by the Clinical Research Coordination Office of the University of Szeged (4430/2018) 7 January 2019.

### 4.2. Immunohistochemistry and Evaluation of Staining Patterns

The IHC reactions were uniformly performed in FFPE sections. Briefly, 2–5 μm-thick paraffin sections were routinely de-waxed, blocked for endogenic peroxidase activities in ethanol containing 1.5% (*v*/*v*) H2O2, and heat-treated in 10 mM Sodium citrate (0.05 % Tween-20, pH 10.0) antigen retrieval buffer using a household electric pressure cooker. After protein blocking in 50 mM Tris-buffered saline (TBS, pH 7.4) containing 5% (*w*/*v*) low-fat milk powder, the sections were incubated with the primary antibodies at room temperature for 70 min. Detection was performed using the Novolink polymer kit (Leica Biosystems/Novocastra, Newcastle Upon Tyne, United Kingdom), and nuclear staining was carried out with Mayer’s hematoxylin. The IHC stains were executed by a four-channel TECAN Freedom Evo liquid handling platform (TECAN, Mannedorf, Switzerland). 

For STX1, we utilized a well-characterized mouse monoclonal antibody HPC-1 (sc-12736; 1:200; Santa Cruz Biotechnology, Dallas, TX, USA), which detects both STX1 A and B isoforms. A tumor sample was considered to be STX1-positive if more than 50% of the neoplastic cells showed either membranous or cytoplasmic staining. The staining intensity was categorized as weak, moderate, or strong. Samples were independently assessed by three of the authors (S.T-N., B.K., and L.K.). In a case with discordant results, a consensus was reached by a second-look evaluation made jointly. 

In NENs investigated using the TMA technique, immunostainings for the most common NE markers, such as SYP (27G12; 1:100; Leica Biosystems), CHGA (5H7; Leica Biosystems/Novocastra, Newcastle Upon Tyne, United Kingdom), and CD56 (MRQ-42; 1:500; Cell Marque, Rocklin, CA, USA), were also performed to compare the results with STX1 expression. In cases where STX1 immunostainings were performed on whole tissue sections, the SYP, CHGA, and CD56 expression was not tested systematically; however, the SYP, CHGA, and CD56 immunostainings performed for the original pathology report were re-evaluated, if available. Cytoplasmic staining for SYP and CHGA or membranous staining for CD56 were considered positive. To identify the various subtypes of gastrointestinal NETs, IHC reactions were performed against VMAT1 (RMT77; 1:100; Leica Biosystems/Novocastra, Newcastle Upon Tyne, United Kingdom) as a marker of EC cells and GLP1 (sc-57166; HYB 147-06; 1:100; Santa Cruz Biotechnology, Dallas, TX, USA) as a marker of L-cells. The sustentacular cell population in paragangliomas and pheochromocytomas was identified with S100.

## 5. Conclusions

In conclusion, we showed that STX1 outperforms common NE IHC markers in terms of its specificity and sensitivity and appears to be the most advantageous immunophenotypic marker of NE cells and NENs. STX1 demonstrated a near-perfect specificity and an outstanding sensitivity, even in NECs. We recommend that STX1 be added to the IHC panel of NE differentiation in routine diagnostic histopathology. The consistent STX1 expression in all NETs, regardless of the anatomical site or subtype, makes it a reliable marker, even in the hands of routine pathologists who are less experienced with NENs and unaware of the specific expression patterns and possible pitfalls of classic NE markers. 

## Figures and Tables

**Table 1:** STX1, chromogranin A (CHGA), synaptophysin (SYP), and CD56 immunoreactivity of NE neoplasms (NENs) and neuroectodermal/neuroepithelial neoplasms.

Gastrointestinal NETs	STX1		CHGA		SYP		CD56	
Gastric enterochromaffin-like (ECL) cell NET	5/5	100.0%	4/4	100.0%	3/3	100.0%	0/1	0.0%
Duodenal non-functioning NET	2/2	100.0%	2/2	100.0%	2/2	100.0%	na	na
Ampullary NET	1/2	50.0%	2/2	100.0%	1/2	50.0%	1/2	50.0%
Small intestinal enterochromaffin (EC) cell NET	12/12	100.0%	12/12	100.0%	12/12	100.0%	8/12	66.7%
Appendix NET	9/9	100.0%	8/8	100.0%	7/7	100.0%	9/9	100.0%
Rectal EC cell NET	2/2	100.0%	2/2	100.0%	1/1	100.0%	0/2	0.0%
Rectal L cell NET	5/5	100.0%	2/5	33.3%	4/4	100.0%	5/5	100.0%
Metastatic NET. gastrointestinal origin	4/4	100.0%	4/4	100.0%	1/2	50.0%	0/2	0.0%
**Pancreatic NETs**								
Pancreatic functioning NET *	3/3	100.0%	2/2	100.0%	2/2	100.0%	1/1	100.0%
Pancreatic non-functioning NET	16/16	100.0%	13/14	92.9%	12/13	92.3%	10/11	90.9%
**Pulmonary carcinoids**								
Pulmonary carcinoid. typical	3/3	100.0%	3/3	100.0%	2/2	100.0%	2/2	100.0%
Pulmonary carcinoid. atypical	2/2	100.0%	na	na	na	na	na	na
**Gastroenteropancreatic NECs**								
Stomach small cell NEC	2/2	100.0%	1/1	100.0%	1/1	100.0%	1/1	100.0%
Duodenal large cell NEC	1/1	100.0%	1/1	100.0%	1/1	100.0%	na	na
Pancreatic large cell NEC	2/2	100.0%	1/1	100.0%	1/1	100.0%	2/2	100.0%
Ampullary small cell NEC	2/2	100.0%	1/1	100.0%	na	na	2/2	100.0%
**Pulmonary NECs**								
Pulmonary small cell NEC	19/19	100.0%	9/10	90.0%	2/2	100.0%	16/16	100.0%
Pulmonary large cell NEC	4/4	100.0%	1/1	100.0%	na		2/2	100.0%
**Etc NECs**								
Merkel cell carcinoma	5/5	100.0%	1/1	100.0%	1/1	100.0%	2/2	100.0%
Medullary thyroid carcinoma	10/10	100.0%	5/5	100.0%	2/2	100.0%	3/3	100.0%
Metastatic small cell NEC with unknown primary	3/3	100.0%	na	na	na	na	2/2	100.0%
Metastatic large cell NEC with unknown primary	3/3	100.0%	3/3	100.0%	na	na	1/3	33.3%
Small cell NEC of the prostate	1/1	100.0%	1/1	100.0%	1/1	100.0%	1/1	100.0%
Small cell NEC of the uterine cervix	1/1	100.0%	na	na	na	na	na	na
Small cell NEC of the breast	1/1	100.0%	na	na	na	na	na	na
Tumors with mixed NE and non-NE components **								
MiNEN **	2/2	100.0%	0/1	0	2/2	100.0%	0/2	0.0%
Mixed tumor with minor NE component **	4/4	100.0%	na	na	2/2	100.0%	0/2	0.0%
**Etc NENs**								
Pituitary adenoma	19/19	100.0%	4/4	100.0%	1/1	100.0%	na	na
Pheochromocytoma/paraganglioma	16/18	88.9%	15/16	93.8%	9/10	90.0%	2/2	100.0%
**Neuroectodermal/neuroepithelial neoplasia**								
Medulloblastoma	8/9	88.9%	na	na	8/9	88.9%	2/2	100.0%
Neuroblastoma	8/8	100.0%	5/5	100.0%	5/5	100.0%	2/2	100.0%
Ganglioneuroma	2/2	100.0%	na	na	na	na	na	na
Ewing sarcoma/PNET	2/2	100.0%	na	na	na	na	na	na

*: 2 insulinomas, 1 gastrinoma. **: the component showing an NE morphology was positive, and the non-NE component was negative for STX1.

**Table 2:** Sensitivity of STX1, CHGA1, SYP, and CD56 in NETs and NECs.

	Sensitivity—NET	Sensitivity—NEC
STX1	99%	100%
CHGA	98%	91%
SYP	96%	89%
CD56	70%	93%

**Table 3:** STX1 immunoreactivity in conventional (non-NE) tumors.

Tumor Type	STX1 (Positive/Total No. of Cases; %)		No. of Cases with Scarcely Scattered STX1 Positive Cells
Colorectal adenocarcinoma	0/8	0.0%	5
Gastric adenocarcinoma	0/12	0.0%	9
Hepatobiliary and pancreatic carcinoma	0/11	0.0%	1
Lung squamous cell carcinoma	0/5	0.0%	
Lung adenocarcinoma	0/8	0.0%	
Head and neck carcinoma	0/7	0.0%	
Basal cell carcinoma of the skin	0/2	0.0%	
Cervical and ovarian carcinoma	0/8	0.0%	1
Prostatic adenocarcinoma	0/7	0.0%	
Melanocytic tumor	0/9	0.0%	
Soft tissue tumor	0/13	0.0%	
Lymphoma and myeloid neoplasm	0/31	0.0%	
Genital germ cell tumor	0/9	0.0%	
Gonadal sex-cord stromal tumor	0/5	0.0%	
Solid pseudopapillary neoplasm (pancreas)	0/1	0.0%	
Adrenocortical adenoma	0/11	0.0%	
Adrenocortical carcinoma	0/12	0.0%	
Parathyroid adenoma	0/19	0.0%	
Papillary thyroid carcinoma	0/5	0.0%	
Thyroid follicular adenoma	0/1	0.0%	
***Carcinomas of the breast***			
No special type	1/18	5.6%	1
Invasive lobular carcinoma	0/2	0.0%	
Mucinous carcinoma, hypocellular type	0/6	0.0%	
Mucinous carcinoma, hypercellular type	7/10	70.0%	
Metaplastic carcinoma	0/1	0.0%	

## Abbreviations

NE	Neuroendocrine
NEN	Neuroendocrine neoplasm
IHC	Immunohistochemistry
SYP	Synaptophysin
CHGA	Chromogranin-A
STX1	Syntaxin 1
NET	Neuroendocrine tumor
NEC	Neuroendocrine carcinoma
IARC	International Agency for Research on Cancer
WHO	World Health Organization
MiNEN	Mixed neuroendocrine-non neuroendocrine neoplasm
NSE	Neuron-specific enolase
PGP9.5	Protein gene product 9.5
INSM1	Insulinoma-associated protein 1
HPC-1	Syntaxin 1
ECL	Enterochromaffin-like
EC	Enterochromaffin
VMAT1	Vesicular monoamine transporter 1
GLP1	Glucagon-like peptide-1
FFPE	Formalin-fixed paraffin-embedded
TMA	Tissue microarray
TBS	Tris-buffered saline
